# Spatial Patterns of Ischemic Heart Disease in Shenzhen, China: A Bayesian Multi-Disease Modelling Approach to Inform Health Planning Policies

**DOI:** 10.3390/ijerph13040436

**Published:** 2016-04-20

**Authors:** Qingyun Du, Mingxiao Zhang, Yayan Li, Hui Luan, Shi Liang, Fu Ren

**Affiliations:** 1 School of Resources and Environmental Science, Wuhan University, 129 Luoyu Road, Wuhan 430079, China; qydu@whu.edu.cn (Q.D.); mxz@whu.edu.cn (M.Z.); li-yayan@whu.edu.cn (Y.L.); 2 Key Laboratory of GIS, Ministry of Education, Wuhan University, 129 Luoyu Road, Wuhan 430079, China; 3 Key Laboratory of Digital Mapping and Land Information Application Engineering, National Administration of Surveying, Mapping and Geoinformation, Wuhan University, 129 Luoyu Road, Wuhan 430079, China; 4 Collaborative Innovation Center of Geospatial Technology, Wuhan University, 129 Luoyu Road, Wuhan 430079, China; 5 School of Planning, Faculty of Environment, University of Waterloo, 200 University Avenue West, Waterloo, ON N2L 3G1, Canada; h3luan@uwaterloo.ca; 6 Shenzhen Prevention and Treatment Center for Occupational Diseases, Guiyuan Street North 70, Luohu District, Shenzhen 518001, China

**Keywords:** ischemic heart disease (IHD), hypertension, Bayesian hierarchical model, multi-disease analysis, Shenzhen

## Abstract

Incorporating the information of hypertension, this paper applies Bayesian multi-disease analysis to model the spatial patterns of Ischemic Heart Disease (IHD) risks. Patterns of harmful alcohol intake (HAI) and overweight/obesity are also modelled as they are common risk factors contributing to both IHD and hypertension. The hospitalization data of IHD and hypertension in 2012 were analyzed with three Bayesian multi-disease models at the sub-district level of Shenzhen. Results revealed that the IHD high-risk cluster shifted slightly north-eastward compared with the IHD Standardized Hospitalization Ratio (SHR). Spatial variations of overweight/obesity and HAI were found to contribute most to the IHD patterns. Identified patterns of IHD risk would benefit IHD integrated prevention. Spatial patterns of overweight/obesity and HAI could supplement the current disease surveillance system by providing information about small-area level risk factors, and thus benefit integrated prevention of related chronic diseases. Middle southern Shenzhen, where high risk of IHD, overweight/obesity, and HAI are present, should be prioritized for interventions, including alcohol control, innovative healthy diet toolkit distribution, insurance system revision, and community-based chronic disease intervention. Related health resource planning is also suggested to focus on these areas first.

## 1. Introduction

Ischemic Heart Disease (IHD) is a leading cause of death worldwide. In China specifically, IHD ranked second of all causes of death in 2012 [1]. According to the Reports of Nutrition and Chronic Disease Status of Chinese Residents in 2015, IHD remains a major cause of death, thus burdening healthcare facilities at all geographic levels. Major risk factors of IHD are hypertension (high blood pressure), smoking, diabetes, lack of exercise, overweight/obesity, high blood cholesterol, high alcohol intake, poor diet, depression, *etc.* However, these risk factors are not readily available in China at small-area levels (e.g., sub-district). Hypertension is the leading cause among all major causes of IHD. Causes of hypertension include overweight/obesity, insulin resistance, high alcohol intake, high salt intake, stress, low potassium and calcium intake, *etc.* Overweight/obesity, and High Alcohol Intake (HAI) are common risk factors shared by IHD and hypertension. In fact, previous studies have shown that IHD risk is positively correlated with hypertension risk [2] and hypertension is responsible for at least 45% of IHD deaths [3]. Therefore, it is possible to use hypertension, which is usually recorded in parallel with IHD, as a proxy covariate for explaining IHD variations.

Interventions including healthcare programs for preventing and controlling IHD have been proven effective since IHD can be easily detected and its causes are well-known [4]. However, along with China’s ever increasing population, healthcare service demand constantly increases. Worse still, the insufficient healthcare resources in China are inappropriately allocated [5]. Spatial (rather than non-spatial) analysis methods investigating geographically varying IHD risks thus could aid the optimization of healthcare resource allocations. Nevertheless, three main limitations exist in extant IHD studies at small-area levels.

Methodologically, traditional non-spatial methods assume that the observations at small areas are identically distributed and independent. This assumption, however, is usually violated in empirical spatial studies. Adjacent areas often have similar, and thus autocorrelated, prevalences of chronic diseases [6]. Accounting for the spatial structures is complex and difficult with conventional frequentist spatial statistical approaches such as spatial error and spatial lag models [7] and is problematic in, for example, estimating risks and capturing overdispersion of count data [8]. The Bayesian hierarchical modelling approach enables researchers to tackle these problems encountered in frequentist approaches and benefits spatial epidemiology modelling at small-area levels. It combines prior information (e.g., expert knowledge and experience) and data information for parameter estimations, and thus is powerful in modelling complex structures of disease risks. Many studies have used Bayesian approaches to map risk patterns of a single disease for intuitive detection. However, few past studies used the Bayesian Multi-disease Analysis (BMDA) approach to analyze IHD and related diseases in the same model. Covariate Model [9] (CM) is a type of BMDA that uses exposure to a second disease as a covariate in the modelling of the first disease. Previous studies have used a lung cancer covariate as a proxy for heavy smoking to analyze the risk of malaria [9], oral cavity, and larynx cancer [10], and chronic obstructive pulmonary disease [11]. Another type of BMDA is the Shared Component Model (SCM) [12], which is an extension of the Besag, York, and Mollié model (BYM) [13]. SCM models relate diseases in a joint formulation, with shared components representing common risk factors. Specifically, the shared components shed light on patterns of multiple relevant diseases, thus naturally and convincingly reflecting common risk distributions [12,14]. Past researches have employed SCM to discover unmeasured risk factors [11,15] and obtained more precise estimates of cancer incidence rates [16]. SCM can also be used to examine the general health status of a region [17] due to its capability to extract common risks of related diseases.

Another limitation is that most previous IHD studies ignored the interaction between IHD and related diseases. In reality, IHD and related chronic diseases sharing common risk factors often co-exist and appear to have an additive effect. It is a fact that IHD and hypertension often co-exist in the same person in China [18]; and the hypertensive patients have twice as high of an IHD risk than the non-hypertensive ones [19]. It has been proven that a multifactorial risk factor intervention strategy considering both blood pressure and cholesterol is more effective in IHD risk control [20]. Usage of hypertension information in IHD study also has a practical advantage, that is, hypertension could be used to explain IHD variations by representing some of IHD behavioral risk factors, such as unhealthy diet or excessive drinking, which are often unavailable at small-area levels. It is suggested that hypertension prevalence patterns should be taken into account in order to investigate IHD risks as it is recognized that IHD studies that are isolated from related Non-communicable Chronic Diseases (NCDs) would be insufficient. Nevertheless, few IHD studies explored such multi-disease interactions.

This paper addresses the two aforementioned shortcomings in previous IHD studies. Specifically, this study aims to: (1) analyze the spatial patterns of the relative risks of IHD at a small-area level facilitated by hypertension information; (2) identify the spatial patterns of common risk factors shared by IHD and hypertension (in particular, these shared risk factors are constructed as a single model component in BMDA models); and (3) propose geographically focused policies for combating IHD in Shenzhen.

## 2. Materials

### 2.1. Study Area

**Figure 1:**
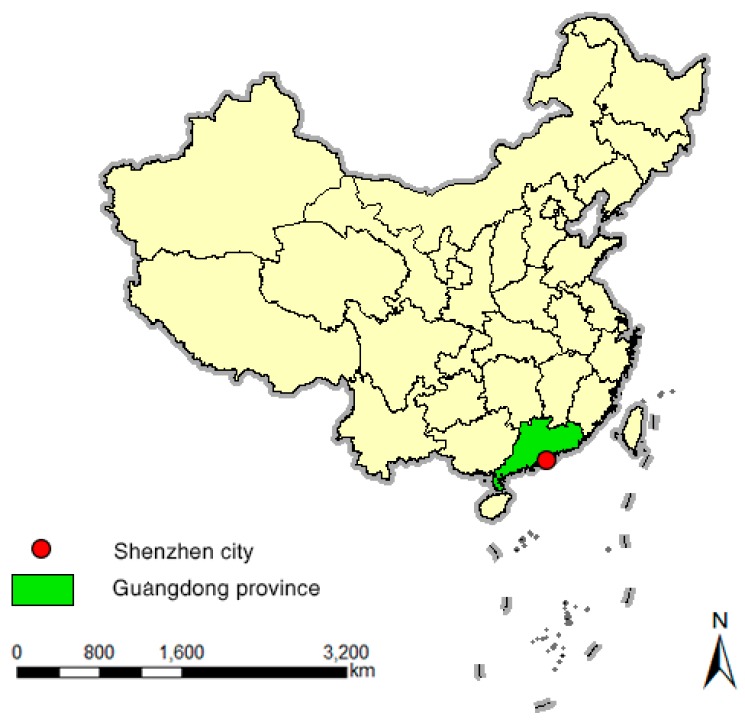
The city of Shenzhen, Guangdong, China.

Our study region, Shenzhen, is a Chinese city located in the southern part of Guangdong province, east of the Pearl River Delta (Figure 1). It is administratively divided into 58 sub-districts (Jie Dao).

### 2.2. Data

**Figure 2:**
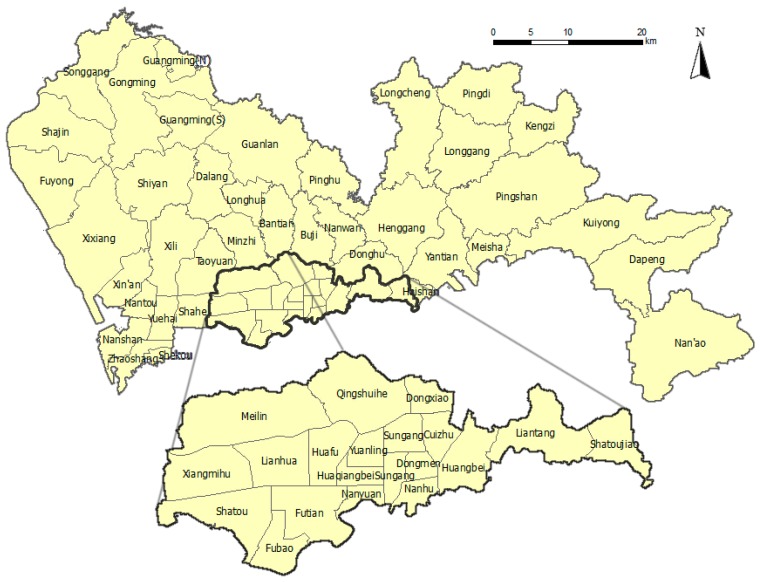
Sub-districts (Jie Dao) of Shenzhen.

The population of each sub-district in Shenzhen was obtained from the 2010 National Census. More precise results could be estimated if the year-matched census data (specifically, the 2012 census) were available. However, the total populations of Shenzhen in 2010 and 2012 were 10.37 million and 10.55 million, respectively [21]. Given the tolerated difference we assumed that the 2010 census data permitted estimation of the parameters to an acceptable degree of precision. Notably, the sub-district of Guangming in the northwest of Shenzhen consists of two parts (Figure 2): Guangming (N) and Guangming (S). Because we only had information on the total population of Guangming instead of each part, we allocated the total population to the two parts according to the proportion of the geographic area each represents.

**Figure 3:**
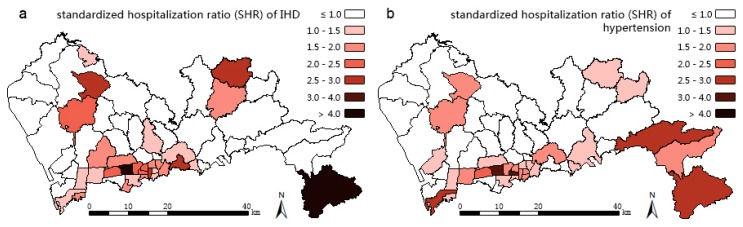
Spatial patterns of standardized hospitalization ratios (SHRs) of (**a**) Ischemic Heart Disease (IHD); (**b**) hypertension in 2012 for Shenzhen at the sub-district level.

The 2012 hospitalization data for IHD and hypertension were collected by the Shenzhen Center for Health Information. IHD refers to I20–I25 from the 10th revision of the International Statistical Classification of Diseases and Related Health Problems [22]; hypertension refers to diseases listed in I10–I15 of ICD-10. The total numbers of hospitalizations for each disease in each sub-district in Shenzhen are used as data in this work. In 2012, 15,256 patients were hospitalized for IHD and 10,395 patients were hospitalized for hypertension in Shenzhen. Figure 3 maps the Standard Hospitalization Ratios (SHRs) of IHD and hypertension. The two maps, along with the relatively high correlation (0.63) between IHD and hypertension at sub-district level, indicate that these two diseases have similar distributions. Specifically, the middle southern region of Shenzhen represents the high SHR center of both IHD and hypertension in Shenzhen in 2012. Both SHRs decrease as one moves outward from this region, and the hypertension SHR pattern extends further in the east-west direction. Notably, the south-eastern sub-districts display exceptionally high SHRs of both diseases because they have very low populations.

## 3. Methods

Bayesian approaches combine prior knowledge (*i.e.*, hypertension could cause IHD, and they have common risk factors) and observed data (*i.e.*, IHD and hypertension hospitalizations) to estimate posterior distributions of unknown parameters (*i.e.*, spatial variations of common risk factors). The development of Markov Chain Monte Carlo (MCMC) simulation methodologies that adopt a Bayesian hierarchical approach has enabled spatial structure to be modelled at the prior level. Three Bayesian hierarchical models were used for our analyses. Model 1 and Model 2 are both covariate models but are different in the way of incorporating hypertension as a covariate, while Model 3 is a shared component model.

### 3.1. Hierarchical Model 1

At the first level, the total number of IHD hospitalizations at sub-district i, $Y_{1i}$ is assumed to follow a Poisson distribution (Formula (1)). The footnote 1 (or 2) denotes the disease type is IHD (or hypertension).

(1) $$Y_{1i}\sim Poisson(E_{1i}\theta_{1i})$$

where $E_{1i}$ is the expected IHD hospitalization number at sub-district i, and $\theta_{1i}$ is the underlying IHD relative risk (RR) at sub-district i. Empirically, the maximum likelihood estimate of RR is the SHR, which is calculated as ${\hat{\theta }}_{1i}=Y_{1i}/E_{1i}$. However, this estimated SHR is flawed with regard to mapping underlying patterns [23]. The second level (Formula (2)) models the relationship between log-RR of IHD and hypertension. α is the intercept, and $u_{1i}$ and $v_{1i}$ are structured and unstructured random effects, respectively. $X_{2i}$ represents the hypertension SHR (= $\frac{Y_{2i}}{E_{2i}}$: $Y_{2i}$ and $E_{2i}$ are the observed and expected hospitalization number of hypertension at sub-district i) and β is the corresponding coefficient.

(2) $$\log(\theta_{1i})=\alpha_{1}+\beta X_{2i}+u_{1i}+v_{1i}$$

### 3.2. Hierarchical Model 2

Unlike Model 1, both the hospitalization numbers of IHD ($Y_{1i}$) and hypertension ($Y_{2i}$) in Model 2 follow Poisson distributions at the first level (Formulas (3) and (4)).

(3) $$Y_{1i}\sim Pois(E_{1i}\theta_{1i})$$

(4) $$Y_{2i}\sim Pois(E_{2i}\theta_{2i})$$

Instead of directly using the hypertension SHR as a covariate, the second level of Model 2 uses the spatially structured random effects of log-RR ($\theta_{2i}$) of hypertension, $u_{2i}$, as a covariate for modelling log-RR ($\theta_{1i}$) of IHD (Formulas (5) and (6)). Since log-RR of hypertension is modelled only as intercepts ($\alpha_{2}$) and structured ($u_{2i}$) and unstructured ($v_{2i}$) random effects, $u_{2i}$ represents the spatial patterns of hypertension.

(5) $$\log(\theta_{1i})=\alpha_{1}+\beta_{1}u_{2i}+u_{1i}+v_{1i}$$

(6) $$\log(\theta_{2i})=\alpha_{2}+u_{2i}+v_{2i}$$

### 3.3. Hierarchical Model 3

Similar to Model 2, at the first level of Model 3, hospitalization numbers of IHD ($Y_{1i}$) and hypertension ($Y_{2i}$) follow Poisson distributions (refer to Formulas (3) and (4)). A shared component $\Phi_{i}$ (rather than spatial random effects of log-RR, hypertension used in Model 2) representing common risk factors of IHD and hypertension was used for modelling log-RR of IHD and hypertension, respectively (Formulas (7) and (8)). $\Phi_{i}$ is assumed to be spatially structured and accounts for spatial structures of IHD and hypertension, together with disease-specific spatial random effects ($u_{1i}$ and $u_{2i}$ for IHD and hypertension, respectively). The coefficients of $\Phi_{i}$ ($\beta_{1}$ and $\beta_{2}$ for IHD and hypertension, respectively) denote the dominating degrees of the shared component in the relative risks of IHD and hypertension. Specifically, $\beta_{1}$ and $\beta_{2}$ are synthesized by a scaling parameter δ [12], where $\beta_{1}$ equals δ and $\beta_{2}$ equals the reciprocal of δ.

(7) $$\log(\theta_{1i})=\alpha_{1}+\beta_{1}\Phi_{i}+u_{1i}+v_{1i}$$

(8) $$\log(\theta_{2i})=\alpha_{2}+\beta_{2}\Phi_{i}+u_{2i}+v_{2i}$$

### 3.4. Prior Specification and Model Assessment

An intrinsic conditional autoregressive (ICAR) [13] distribution was assigned to the spatially structured random effects ($u_{1i}$ in Model 1; $u_{1i}$ and $u_{2i}$ in Models 2 and 3) and shared component $\Phi_{i}$ (in Model 3). ICAR has been widely used as priors for spatial parameters in Bayesian modelling. Using ICAR, the means of $u_{1i}$, $u_{2i}$, and $\Phi_{i}$ depend on the adjacent $u_{i}$s, $u_{2i}$s, and $\Phi_{i}s$. Variance parameters $\sigma_{u_{1}}^{2}$, $\sigma_{u_{2}}^{2}$, and $\sigma_{\Phi }^{2}$, which are inversely proportional to area *i*’s number of neighbors, were specified for controlling variabilities of $u_{1i}$, $u_{2i}$, and $\Phi_{i}$, respectively. A highly vague prior Gamma(0.001,0.001) was specified for the reciprocals of $\sigma_{u_{1}}^{2}$, $\sigma_{u_{2}}^{2}$, and $\sigma_{\Phi }^{2}$ (denoted as $\tau_{u_{1}}$, $\tau_{u_{2}}$, and $\tau_{\Phi }$), respectively. Priors for the intercept ($\alpha_{1}$ in Model 1, and $\alpha_{1}$ and $\alpha_{2}$ in Models 2 and 3) are improper uniform distribution on the whole real line. Unstructured random effects ($v_{1i}$ in Model 1, and $v_{1i}$ and $v_{2i}$ in Models 2 and 3) were assigned a prior of normal distribution with mean 0 and variance $\sigma_{v_{1}}^{2}$ (or $\sigma_{v_{2}}^{2}$). Similarly, Gamma(0.001,0.001) was assigned as priors to the reciprocals of $\sigma_{v_{1}}^{2}$ and $\sigma_{v_{2}}^{2}$ (denoted as $\tau_{v_{1}}$ and $\tau_{v_{2}}$), respectively. Priors for the coefficients ($\beta_{1}$ in Models 1 and 2) displayed a vague normal distribution with mean 0 and variance 1000. The prior for δ displayed a log-normal distribution with mean 0 and variance $\kappa_{\delta }$. Specifically, we assigned 5.88 to $\kappa_{\delta }$, which has been used previously [17].

Each of the three models was fitted with three chains in WinBUGS 1.4 [24] using Markov Chain Monte Carlo (MCMC) simulation. We monitored chain convergence by checking the history plots, trace plots, autocorrelation plots, and Gelman-Rubin plots. After convergence, the three chains of each model were run for another 250,000 iterations, giving 300,000 (= 3*100,000) samples in total for posterior estimations. Monte Carlo errors of parameters of interest in each model reached acceptable levels (<5% sample posterior deviation). Model fit was assessed with the Deviance Information Criterion (DIC) [25]. Models with smaller DICs are considered better fit the dataset.

## 4. Results

**Figure 4:**
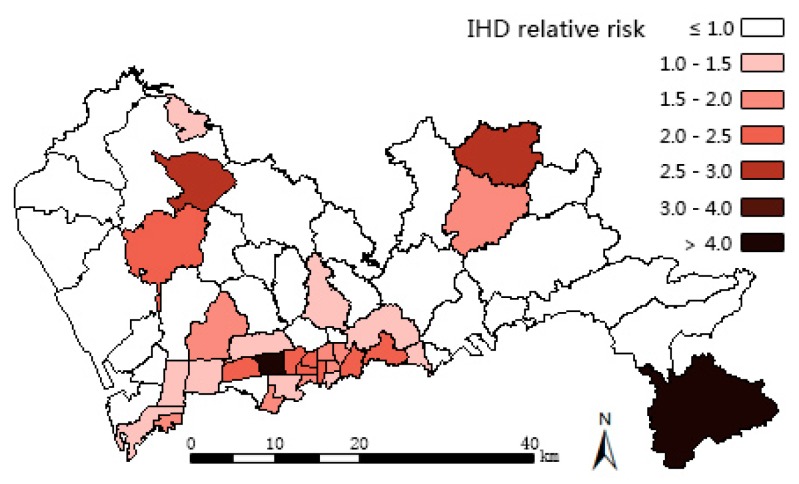
Results obtained using Model 1. Spatial patterns of posterior estimates of overall IHD relative risk (RR).

**Figure 5:**
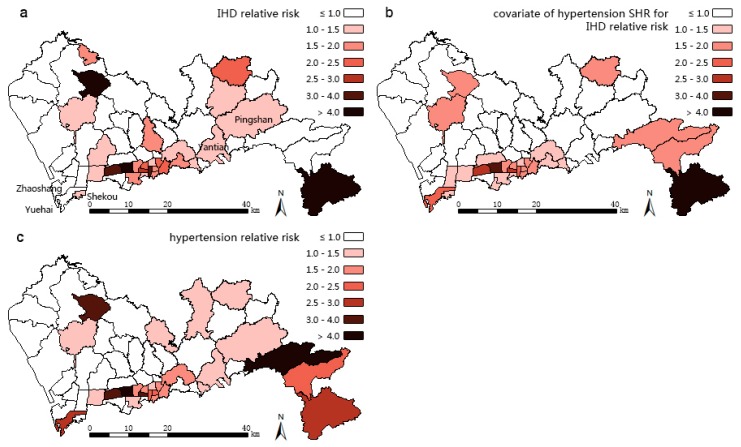
Results obtained using Model 2. Spatial patterns of posterior estimates of (**a**) overall IHD relative risk (RR); (**b**) RR of IHD explained by hypertension; (**c**) overall hypertension RR.

**Figure 6:**
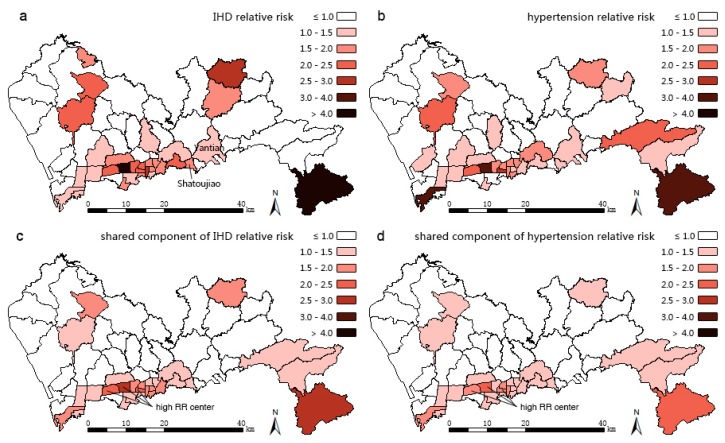
Results obtained using Model 3. Spatial patterns of posterior estimates of (**a**) overall IHD relative risk (RR); (**b**) overall hypertension RR; (**c**) shared component (with coefficients) of IHD; and (**d**) shared component of hypertension RRs.

The results of three models are mapped in Figure 4, Figure 5 and Figure 6. Compared with the IHD SHR pattern (Figure 3a), the IHD RR pattern (Figure 4) of Model 1 makes almost no difference. The IHD RR pattern of Model 2 (Figure 5a) is much more polarized; the sub-districts of Pingshan and Yantian (northeast of the high SHR center) were estimated to have potentially higher IHD hospitalization rates while Zhaoshang, Yuehai, and Shekou (southwest corner of Shenzhen) appear to have potentially lower rates of hospitalization. The high RR center in Figure 5a moves slightly northeastward compared with the IHD SHR pattern in Figure 3a. The IHD RR pattern of Model 3 (Figure 6a) shows a few changes, suggesting that Yantian and Shatoujiao would have potentially higher IHD hospitalization rates, while Zhaoshang would have a lower IHD rate. Similar to the results from Model 2, Figure 6a also shows that the geographic center of IHD hospital admissions in Shenzhen exhibited a slight northeastward shift. The results obtained from the three models consistently show that the potential IHD risk develops in the same directions. Comparatively, Model 2 outperforms Models 1 and 3 in providing more details; its risk surface spans more gradually, presenting clearer disparity.

Table 1 reports the results from the three models. Model 2, which has the lowest DIC (488.6), is statistically better than both Model 1 (530.2) and Model 3 (520.8). The component ratio denotes the proportion of spatial variation of IHD that is captured by hypertension (covariate part of Model 1), part of hypertension (covariate part of Model 2), or common risk shared with hypertension (shared component of Model 3). The usage of the component ratio is inspired by Best and Hansell [11], who were interested in differentiating components contributing to the risk variations between models. In our study, not accounting for measurement error, the ratio of the covariate part of Model 2 is 72.5%, lower than that of Model 1 (85.5%) but higher than the shared component ratio of Model 3 (69.6%), this suggests that the spatial structured part of hypertension captures less IHD variations than the whole hypertension, but more than the shared term. Model 3 is intended to extract the patterns of common risk factors that we assume to be HAI and overweight/obesity risks (see Section 1), and the two extracted patterns of the shared components with disease-specific coefficients for IHD and hypertension are shown in Figure 6c,d respectively. In both patterns, high common risk clusters in the middle south of Shenzhen and spreads in the east-west direction. The five sub-districts of Lianhua, Huaqiangbei, Yuanling, Xiangmihu, and Guiyuan stand out, showing excessive relative risks exceeding 1.50, thus, they become the cluster centers. Comparing Figure 6c with Figure 6a,d with Figure 6b, it can be seen that these centers also turn to be high IHD RR center and the high hypertension RR center.

## 5. Discussion

### 5.1. BMDA to Discover Spatial Patterns of Disease

There have been many approaches to predict diseases [26], such as using complex networks to explore comorbidity relations among chronic diseases in order to identify future risk of diseases [27]. BMDA, which has been applied here, is also a methodology to analyze multiple chronic diseases together for an NCD integrated intervention strategy in China. It is pathologically convincing to present the potential risk of a disease by modelling its causationally related disease as a covariate in BMDA. BMDA is superior to traditional spatial regression explanatory risk factors dealing with multiple explanatory risk factors in two aspects. Firstly, BMDA takes certain risk factors as a whole as a disease (*i.e.*, hypertension). Hypertension as a whole is shown to better at explaining IHD risk rather than the separated basic risk factors, which can also be seen in the component ratio comparison in Table 1: the contribution of the shared component to the IHD overall RRs (69.9%) is less than those of the covariates (85.5% and 72.5%). This implies that hypertensive risk factors may react and function with respect to each other and the improper structuring may lead to false inferences. Hypertensive risk factors have been shown to be additive [28]. Moreover, some risk factors, such as smoking, heavy drinking, and poor diet, co-exist and cluster in specific groups of people, and changing one factor could influence the others [29]. Secondly, BMDA also models unknown risk factors as spatial structured random errors to avoid overestimations, which is a shortcoming of traditional spatial models [7]. Spatial error modelling in BMDA could lead to meaningful findings. In our study, as in most other studies, the covariate component and the shared component are the intended parts in CM and SCM with explicit representations. However, the part of RR that is not explained by CM or SCM is simulated as spatially structured or unstructured errors, which are also informative in that they may represent unknown factors that contribute to RR variations [30]. For example, Best and Hansell [11] utilized the error component to reveal additional risk factors and discovered higher chronic obstructive pulmonary disease mortality in conurbation and mining areas, a factor that is historically associated with heavy industry and higher air pollution levels. In our paper, we only estimated the ratios of error components to evaluate how efficiently the model facilitates the hypertension data. However, error patterns may inspire further discoveries of certain underlying socioeconomic or psychological risk factors of IHD, which are not fully proved yet by traditional methods [31]. Adding structured error components as covariates to the model facilitates decreasing the residual spatial structure and all or most structured risk factors could be explored in this way [30].

### 5.2. BMDA to Discover Spatial Patterns of Unmeasured Risk Factors

BMDA, as used in this paper, is an efficient methodology to explore unmeasured common risk factors and thus could complement the current Chinese disease surveillance system. The Chinese Center for Disease Control and Prevention (Chinese CDC) established the Chronic Diseases and Risk Factors Surveillance Survey (CDRFSS) system in 2004 to examine the prevalence of essential behavioral risk factors of NCDs among registered residents. Data from CDRFSS are intended to support multiple chronic disease integrated prevention and advanced scientific research. However, these data are incomplete, inconsistent, inaccurate, and often lagging behind, which is attributable to the lack of small-area level data [32], proper statistical models [33], and uniform and scientific specifications [34], as well as the need for arduous manual data entry [35] and lack of ignorance of unregistered floating population. Spatial patterns of NCD risk factors discovered with BMDA could be used as surrogates of unmeasured small-area level risk factors, thus supplementing CDRFSS databases to benefit related NCD integrated intervention. In this paper, we assumed that the common risk factors shared by IHD and hypertension are overweight/obesity and HAI. In order to confirm their spatial variations of common risk factors shown in Figure 6c,d, data of these risk factors should be obtained and accounted for in the statistical model (either as covariates or as additional “diseases”).

### 5.3. Policy Applications

In order to promote the effectiveness of integrated IHD prevention it has been emphasized that there is a need to focus on the hypertension intervention in the whole cardiovascular disease prevention and control [4]. The middle southern region of Shenzhen has high risks of both IHD and hypertension (Figure 4, Figure 5a,b), thus requiring immediate interventions. While the middle southern parts have already been addressed with critical risks [36,37], the northeast part should be focused upon next due to its potential high risks, according to our findings. In these areas, common community-based integrated NCD intervention measures should be strengthened including: (1) diet: restriction of sodium intake to no more than six grams per day, encouragement of lower protein and fat intake, and prohibition of smoking and alcohol use; (2) exercise: personalized schema of jogging, walking, or Tai Chi and outdoor activities, especially for obese people; (3) psychology: guidance in achieving a pleasant and stable mood; and (4) medicine: instruction in proper medicine use and popularization of health knowledge in various ways, such as via dissemination outlets or notices posted on bulletin boards. Such community-based directions in Shenzhen have been confirmed to be effective in the control of hypertension [38] and in reducing the economic burden on residents [39]. However, the current NCD intervention system needs improvement. For example, proper policies are called for to promote the practice of the Chinese Dietary Guidelines and the Food Pagoda [40] and multi-sector collaboration, and cover insurance for the uninsured 43.1% floating people in Shenzhen [41]. At the community level, the professional capacity of community healthcare staff should be increased by providing preferential training in areas with the most need [42]. The electronic medical records of resident behavioral risk factors should also be further analyzed and utilized. In addition, to achieve better results in these high IHD and hypertension risk sub-districts, the local community healthcare centers could first try to follow the policy entitled “hypertensive patients free to have essential drugs and standard formula treatment” proposed by Wang Longde [4].

Findings from Model 3 (the shared component model) show that common risk factors contribute most to IHD, implying that the common risk factors, which are assumed to be overweight/obesity and HAI, should be the primarily areas to receive intervention. To help residents maintain healthy weight, residents should receive innovative family or personal toolkits which include rulers for measuring waist and BMI, limited salt spoon, and a limited oil pot; additional materials consisting of instructions from doctors, dietary records, and balanced diet charts should be handed out to the residents in order to increase their self-knowledge about health conditions. These intervening tools are intuitive, quantitative, and personalized, and thereby should assist with increasing the residents' compliance. As an appropriate strategy, the toolkits should be preferentially provided to the less educated population, while for the more educated segment of the population, self-management is instead advocated [43]. These toolkits and instructions could be directed towards the high overweight/obesity and HAI risk regions in Shenzhen (Figure 6c or Figure 6d). Alcohol control issues should also receive increased social attention there. Population-based early-stage prevention of HAI should be further developed [44]. Additionally, the reliable and valid alcohol screening method Alcohol Smoking and Substance Use Involvement Screening Test (ASSIST) should be promoted in these sub-districts [44,45]. Residents could be directed towards the Chinese Dietary Guidelines [46] which suggests that an adult male should not intake more than 25 g of alcohol per day, and that an adult female should intake no more than 15 g of alcohol per day. HAI-specific treatment facilities and family-based alcohol rehabilitation centers should be built [47], and some related self-help organizations should be directed to cover these high risk areas in Shenzhen.

### 5.4. Implications for Planning

Spatial patterns of common risk factors, overweight/obesity, and HAI reveal varying potential NCD burdens and healthcare service needs across space, which is essential information to support spatial allocation or adjustment of healthcare resources. Our preliminary studies of hospital accessibility and inpatient behaviors identified that the actual burden of hospitals in the middle southern part of Shenzhen (the metropolitan area) is relatively higher than that in the northern part, even though the best eight hospitals are all located in the middle southern part. This is due to the fact that the patients living all over Shenzhen have a tendency to travel a long distance to get hospitalized in these central hospitals. With high IHD risk found in this paper, the middle southern sub-districts including Lianhua, Huaqiangbei, Yuanling, *etc.*, are severely challenged by both high disease burden and high healthcare resource burden. Three strategies can be proposed to alleviate such burdens. One is to strengthen the professional ability of community healthcare for NCD chain-service all over Shenzhen to obtain residents’ trust in primary healthcare, but the task is arduous and would have a long way to go. The second strategy is to allocate more healthcare resources, especially specialized NCD hospitals to share the burden of the general hospitals [48]. The last strategy is to establish higher class hospitals in the non-central part of Shenzhen in order to decentralize the patients, which is already on the planning agenda of Shenzhen [48]. According to The General Office of the State Council of China [49], the establishment of cross-regional public healthcare services in regions such as Pearl River Delta where the conditions for regional integrated development exist could be explored. Shenzhen has always been an innovative city, and now it is being encouraged to remove the restrictions imposed by administrative divisions to plan cross-district healthcare service facilities based on overall considerations. The accurate evaluation of potential healthcare demands over space, represented by NCD risk factor patterns in this paper, would benefit the realization of the overall healthcare planning in Shenzhen.

### 5.5. Limitations of the Study

Our study has several limitations. First, the boundaries of Shenzhen restrained the outer sub-districts from making use of their neighbor’s information in regions other than Shenzhen in our models. For example, Nan’ao has only one neighbor in Shenzhen, and because its population is very small, its abnormally high risk was not substantially improved by the models. This problem can be avoided by enlarging the study area, and refining the divisions. Second, although HAI and overweight/obesity are two different risk factors, they were studied in combination as a result of insufficient information being available to allow us to make a distinction. Third, the use of IHD and hypertension data to identify the pattern of HAI and overweight/obesity limited the integration of information regarding other very different types of diseases related to alcohol and weight issues, such as hepatitis and liver cancer. In the future, the patterns discovered here could be verified and adjusted using such data. The use of diverse disease data also permits the study distinguish between HAI and overweight/obesity.

## 6. Conclusions

We presented a Bayesian multi-disease approach involving pathogenic disease of IHD (*i.e.*, hypertension) to explore the spatial patterns of IHD hospitalization rates by CMs in Shenzhen, and further applied an SCM to extract patterns of unmeasured HAI and overweight/obesity risk factors. This paper has two main contributions. First, findings in terms of IHD relative risk patterns highlight sub-districts where health planning policies and/or IHD prevention programs could be geographically focused. Second, the innovative application of SCM modelling extracted the unmeasured spatial patterns of overweight/obesity and HAI at the sub-district level and thus could be a suitable supplement to the present CDRFSS system in China. The BMDA approach is especially useful in NCD research, and China calls for such advanced methodology to support the NCD integrated intervention work, though it has significant room for improvement. With the use of larger regions and additional data, Bayesian multi-disease analysis could be used to address more interesting and complicated phenomena. Shenzhen is an open, innovative, and progressive city that is a leader in Chinese informationization, construction, and urban planning. Our work can be used to benefit advanced NCD management, direct policymaking regarding problems related to alcohol consumption and obesity/overweight issues, and, finally, support healthcare resource planning in Shenzhen.

## Figures and Tables

**Table 1:** Variances in log-form relative risks of IHD and hypertension; ratios of components in relative risks; and Deviance Information Criterion (DICs) of models.

Model	Disease	Parameter/Component	Median CI (5%, 95%) of Variances	Median CI (5%, 95%) of Component Ratio	DIC
1	IHD	$\beta_{1}$	0.789 (0.517, 1.046)	-	530.2
		$\beta_{1}\frac{Y_{2}}{E_{2}}$	0.708 (−6.318, 4.480)	85.5% (75.0%, 85.6%)	
2	IHD	$\beta_{1}$	1.494 (0.790, 2.502)	-	488.6
		$\beta_{1}u_{2}$	0.014 (−0.130, 0.132)	72.5% (41.6%, 80.2%)	
3	IHD	$\beta_{1}$	1.145 (0.873, 1.494)	-	520.8
		$\beta_{1}\Phi$	−0.023 (−0.120, 0.121)	69.6% (44.3%, 78.4%)	
	hypertension	$\beta_{2}$	0.873 (0.669, 1.145)	-	489.1
		$\beta_{2}\Phi$	−0.017 (−0.091, 0.092)	80.2% (49.2%, 90.6%)	
